# The systemic and local interactions related to titanium implant corrosion and hypersensitivity reactions: a narrative review of the literature

**DOI:** 10.1186/s40729-024-00578-3

**Published:** 2024-11-22

**Authors:** Alexander Swalsky, Sammy S. Noumbissi, Thomas G. Wiedemann

**Affiliations:** 1 New York University - College of Dentistry, 345 East 24th Street, New York, NY 10010 USA; 2 International Academy of Ceramic Implantology, Università degli Studi “G. d’Annunzio” Chieti-Pescara, Chieti, Italy; 3 Department of Oral- and Maxillofacial Surgery, New York University—College of Dentistry, 345 East 24th Street, New York, NY 10010 USA

**Keywords:** Metal allergy, Titanium corrosion, Titanium systemic effects, Tribocorrosion, Titanium implant failure, Zirconia-based implants, Titanium intolerance

## Abstract

Both commercially pure titanium and titanium alloys are established biomaterials for implantation in bone and are widely used today in dentistry. Titanium particulates have been shown in some patient clusters to induce cellular immune mediators responsible for type I and IV hypersensitivity reactions, causing amplified corrosion, osteolysis, and increased odds of implant failure. Systemically, titanium particles were found to affect varying organ tissues and cause potentially harmful effects. In vivo and vitro studies have shown that titanium dental implant corrosion can be induced by factors relating to bio-tribocorrosion. In this literature review, the consequences of titanium implant corrosion and particulate dissemination are discussed and later juxtaposed against a promising novel implant material, zirconia. Zirconia offers characteristics similar to titanium along with additional advantages such as being non-corrosive and having a lower propensity for inducing immune responses. From the mounting evidence discussed in this article, metal allergy testing would be advantageous for choosing an appropriate implant material to minimize potential adverse effects on cellular functions of local and diffuse tissues. Objective: This literature review aims to elucidate and describe mechanisms in which titanium implants may become corroded and induce cellular aberrations both locally and systemically in vivo. Implications of this study provide supportive evidence regarding the selection of appropriate biomaterials for implant patients susceptible to mounting a hypersensitivity reaction to titanium.

## Introduction

Titanium (Ti) is a transitional white metal that was first isolated in 1910 and has since been applied for multiple purposes [1]. Approximately 95% of utilized titanium-containing materials are of an oxidized form; titanium dioxide (TiO2/TiO). Some uses for TiO2 include pigments for paint, whiteners, paper production, cosmetics, and toothpaste due to its opaque coloration and stability under UV light [1]. The remaining 5% of titanium is fabricated for biomaterials due to its desirable characteristics of being typically biocompatible, having a propensity for osseointegration, superior strength against occlusal forces, corrosion resistance, and non-magnetic properties [1, 2]. Titanium implants can be categorized into two. distinct groups; commercially pure (CP) and titanium-based alloys. Commercially pure titanium however still contains trace elements such as carbon, oxygen, nitrogen, and iron which quantitatively increases from grade I to IV leading to grade IV being the strongest and least ductile [3]. Two titanium implant alloys are commonly utilized; Ti-6Al-4 V and Ti-6Al-4 V-ELI (extra low interstitial alloys). The crystalline structures found in these alloys can vary in composition of alpha (α), beta (β) as well as alpha–beta and originate depending on production methods [3]. The alpha crystallographic structure is highly packed and hexagonal while beta is a body-centered cubic form. With the use of aluminum or vanadium, the alloys become closer to the modulus of elasticity of bone (compared to CP titanium) as well as less dense of material and more resistant to corrosion [3]. Although the titanium materials mentioned have great attributes which is evident by their reliable success rates, there has been mounting scrutiny due to their contents having a propensity to negatively impact local and systemic tissues due to immune hypersensitivities, reactive oxygen species (ROS), and corrosion reactions in select groups of patients. This literature review aims to describe and characterize some of the impacts Ti-based dental implants can have on systemic tissues, and peri-implant tissues as well as assess how viable zirconia-based implants can be as an alternative material for implantology.

## Methods

For data and evidence sequestration, electronic databases such as Pubmed/Medline, EMBASE, Cochrane Library, and Google Scholar were utilized. Keywords such as “metal allergy”, “titanium corrosion”, “tribocorrosion”, “titanium hypersensitivity”, “immunological response to titanium”, “zirconia implants”, “titanium implant healing”, “systemic responses to titanium” and “implant osseointegration” were used to narrow down articles to match relevant material. The inclusion criteria were articles written only in the English language, referenced authors, and journals such as The Journal of Oral Ceramic Implantology, Materials, Nanotheranostics, International Journal of Dentistry, Journal of Periodontal Research, Implant Dentistry, and Journal of Proteomics. The initial literature results consisted of 76 articles ranging from 1997 to present. The resulting selection and exclusion of articles was done based on relevance to the subject of interest.

## Discussion

### Particle formation and corrosion

The observed biocompatibility of titanium implants can be attributed to the formation of a passive oxide layer. However, when the oxide layer is lost, it leads to increased corrosion rates [4, 5]. It is important to note that corrosion and particulate dissemination are continuous and independent processes that may synergize to produce amplified consequences [6]. *Pettersson *et al. noted a significant immune response to Ti ions in solution compared to small titanium particulates, suggesting Ti ionization is a key factor in inducing inflammatory responses [7]. Particulate production comprised of titanium and titanium ions can be noted just hours after implant placement, being detected in the peri-implant sites due to mechanical wear of the surface [8]. In terms of prolonged presence, peri-implant tissues were noted to contain larger quantities of titanium particles with reported concentrations being between 100 and 300 ppm [4, 5, 9]. These findings suggest that during the initial implant placement, the surface TiO2 layer will shed exposing and leaving implant surfaces vulnerable to corrosion attack.

### Tribocorrosion

The term *tribocorrosion* is used to describe the influences of mechanical force, and chemical and electrochemical interactions that induce the surface breakdown of dental implants [1, 10–12]. Mechanically induced particulate formation and surface wear can be attributed to functional stressors, micromovements termed fretting as well as surface wear from peri-implant prophylaxis during home care or hygiene visits. These mechanical factors disrupt the passive oxide layer integrity and expose the subsurface of the implant leading to increased corrosion vulnerability [2, 10]. Modes of corrosion have been described by *Noumbissi *et al*.* stating that the most common types of wet corrosion occurring in the oral cavity are galvanic, pitted, and crevice. Significant consideration has been given to reducing galvanic corrosion as it is of the three wet corrosions mentioned, the most prevalent [6, 10, 13]. This corrosive process occurs when alternate metallic materials transfer electrons via direct contact or by a galvanic cell formation due to tissue and saliva electrolyte bridging [3, 6, 10]. Pitting corrosion is due to functional forces causing mechanical wear and is often observed at the prosthetic abutment interface [10]. Like pitting corrosion, crevice corrosion often occurs at the abutment-implant interface where the contacts are hypoxic. Along with mechanical and the three wet corrosion reactions mentioned, immunologic processes significantly influence titanium corrosion and particulate dissemination. These immunologic considerations will be further elaborated on in the following sections.

### Reduction in pH and exogenous corrosion factors

In many circumstances, intra-oral and tissue pH can be lowered, increasing corrosion and tissue titanium particle levels. Some circumstances that lower pH are wound healing, immune responses as well as exogenous origins; bacterial metabolism, fluoride usage, and other chemicals as demonstrated by *Kotaskis *et al. [5, 10, 12–16]. Previous studies have exhibited bacterial adhesion affinity to titanium implant surfaces and furthermore, when titanium implants were debrided with antimicrobials such as chlorhexidine, alterations to the implant surface as well as biocompatibility were affected [3, 12, 13, 16]. When anaerobic microbes are present, their metabolic processes produce compounds such as MnO2, FeCl2, MnCl2, Fe2O3, and FeO which favor corrosion and the production of microgaps on the metallic surfaces such as around the abutment site [10, 13]. Periodontal pathogens such as Porphyromonas gingivalis have been shown to produce corrosive metabolic products such as lipopolysaccharides (LPS), Hydrogen Sulfide (H2S), Methanethiol (CH3SH), and Dimethyl Sulfide ((CH3)2 S) [5, 12]. Although fluoride successfully reduces the risk of caries as well as inhibiting microbial metabolism, it has also been shown to reduce salivary pH, further inducing the loss of corrosion resistance on titanium implant surfaces, and ultimately producing titanium ion dissemination [5, 10, 13]. For these reasons, evidence points towards user cessation of fluoride-containing gels and greater utilization of less abrasive toothbrushes [10].

## Cellular immune responses to titanium

The immune system is markedly responsible for the initiation of peri-implant bone loss.

As a consequence of titanium corrosion and particulate production, inflammatory cells may take up this debris by methods of diffusion or endocytosis [6, 13, 17, 18]. This section will discuss titanium implant particulate influence over immunomodulatory cells.

### Internalization of foreign metal

It has been established that there is an inverse relationship between increased biological activity and decreasing particle size due to greater overall surface area and atom exposure once oxidation of the surface has occurred through processes involved in phagocytes such as macrophages [1, 6, 19]. There are a few determined means of intracellular titanium incorporation such as diffusion, phagocytosis, pinocytosis, and cellular membrane disturbances. Diffusion of titanium particles occurs when the diameter is 2 μm. These particles are recognized to be readily diffused in neutrophils and macrophages resulting in inappropriate recruitment of inflammatory cells to surrounding tissues [8]. *Sansone et a*l. described endocytic receptors as having an affinity for particulates around 50 nm while smaller particles were additionally taken up via pinocytosis. Pinocytosis is another form of endocytosis that can be split into four unique mechanisms of intake which could be compared by vesicle sizes; macropinocytosis (vesicle size > 1 μm), clathrin-mediated endocytosis (120 nm), caveolae-mediated endocytosis (60 nm) and clathrin/caveolae independent endocytosis (90 nm) [20]. *He *et al. noted titanium nanoparticles to be taken up by both clathrin and caveolae-mediated endocytosis. These findings are further supported by caveolae-mediated endocytosis occurring within liver cells in animal studies [20–22]. To conclude, titanium dioxide nanoparticles were deemed to utilize means of pinocytosis in human cell models, such as cervical cancer cells (HeLa), prostate cancer cells (PC-3 M), and osteoblast cells [20, 23, 24]. These findings suggest one mode of titanium particles' initiating cellular responses. Research done by *Soloviev *et al. found that titanium dioxide particles could activate macrophages without initial phagocytosis. Titanium dioxide in this study was found to generate free radicals that lead to peroxidation of the plasma membrane and activation of sphingomyelinases [19].

### Cell responses to titanium nanoparticle debris

**Fig. 1:**
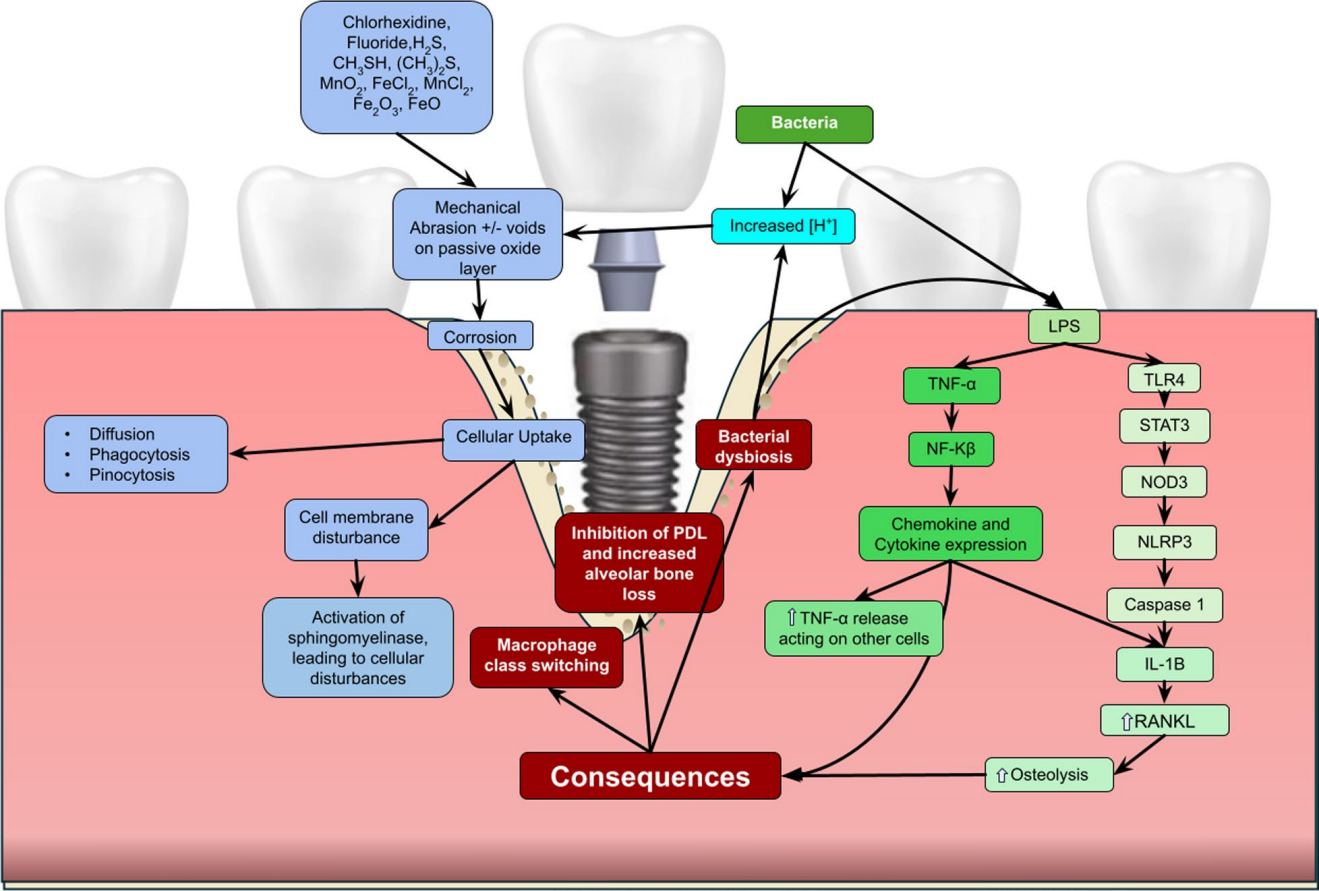
Summary of Corrosion process and particulate infiltration into affected peri-implant cells

Both types I and IV hypersensitivity reactions have been noted in cluster patients who had failed titanium-based dental implants [1, 4, 11]. A group of immune cells known as antigen-presenting cells (APCs) are intimately involved and responsible for both innate immune responses as well as Type I and IV hypersensitivity reactions (Fig. 1). Type I hypersensitivity reactions are by nature more acute, being substantially composed of leukocytes occurring within hours of an insult leading to degranulation and release of histamine from mast cells by IgE. This release of IgE vasodilates vessels and permits edema which is seen in food allergies. Intrinsically, hypersensitivity type IV reactions are of delayed response implying its relevance for appraisal in prospective cases of titanium implant failure. Local tissues such as endothelial cells when exposed to titanium nanoparticles exhibited upregulation of p-selectin ICAM-1, VCAM, and CD44, attracting cells to affected sites such as monocytes, lymphocytes, granulocytes, and macrophages [25]. Resident APCs such as dendritic cells were noted to have both selectively decreased chemokines and costimulatory molecules to reduce MHC II presentation [26]. Notably, however, CCR4 was increased among titanium nanoparticle introductions suggesting specific cellular responses to titanium nanoparticles [1, 10, 26]. Proteomic changes are noted once titanium nanoparticles are intracellular. *Sund *et al. found that these nanoparticles caused proteins involved in metabolic homeostasis, cytoskeleton remodeling, and oxidative stress to have become notably altered with cytosolic protein acetylation [27]. In conjunction with this, phenotype changes, degenerative impacts, and mutation were observed in macrophages and neutrophils along with predominant class switching of macrophages to M1 [8, 28, 29]. Innate cell responses are expected when foreign insults are detected. Toll-like receptors (TLR) mediate cellular signals, inducing inflammatory and adaptive responses. In the case of titanium nanoparticles, TLR4 was found to be variably upregulated due to the presence or absence of LPS ultimately increasing mRNA levels of TNF-α, IL-1β, and IL-6 [4, 10, 30, 31]. Signal transducer and activator of transcription protein 3 (STAT3) act as upstream transcription factors involved in innate cellular immune responses such as activation of NOD-like protein 3 (NLRP3) which subsequently activates caspase-1, leading to the release of IL-1β [7, 32–34]. NLRP3 was found to be associated with both septic and aseptic (titanium-induced) periapical lesions [33]. This association can be shown as lysosomes attempt to degrade titanium particles, releasing cathepsin b which acts as a damage-associated molecular pattern (DAMP), activating NLRP3 [7, 12]. Dendritic cells were noted to display lessened MHC II and co-stimulatory molecules such as CD40, CD80, and CD86 along with chemokine receptors (CCR) CCR6, and CCR7 [26]. From the studies mentioned, titanium dioxide particles impact many cellular components, causing immunomodulation and inflammation.

## Local tissue responses to titanium implants

### Healing and peri-implant biological interface

To reiterate, titanium implants have been historically shown to have desirable healing capacities within tissue and therefore are seen as a standard. Along with being hydrophilic and having high wettability, modifications to implant surface topography, as well as thread design, are some of the methods taken advantage of to optimize and mitigate implant complications leading to failure. A key focus of interest for analysis is on resident tissue responses to implants without resorting to retrieval in cases of successful implantation. The utilization of implant healing caps and abutments has been shown to prove a useful mode of tissue retrieval for histological analysis and to assess implant modification viability [35, 36]. In a particular study, immunohistological analysis of titanium implants done by healing cap removal displayed local infiltrates specific to wound healing; consisting of vascular endothelial growth factor (VEGF), ki 67 expression, nitric oxidase (NOS 1&3), inflammatory cell mediators such as lymphocytes, histiocytes, plasma cells and granulocytes [35]. In regards to implant threaddings, *Valente *et al*.* found bone collagen fiber orientations (BCFO) were of a more transverse orientation at threaddings in the lower flanks while longitudinal were more abundant in narrow threaddings as well as in wider inter-thread spaces [17].

#### Implant failure

Implant failure can be caused by many factors that synergize to result in fracture of the implant body and bone loss. Although not common, implant bodies may fail due in part to flaws in manufacturing, design, and or improper situating of the implant leading to exacerbation from biomechanical actions [3]. In conjunction with the implant body flaws, tribocorrosion may synergize to facilitate crack propagation which has been reported in titanium alloys [3, 10].

#### Ion release and ROS production leading to aseptic osteolysis

Aseptic osteolysis can be defined as the loss of bone by biological processes without the direct influence of bacteria. As previously stated, titanium ion release is a continuous and multifactorial process influenced by various factors. Regardless of the mode of corrosion and ionization, degenerative cellular impacts will occur with local increased expression of TNF-α, IL-1β, IL-6, IL-8, and RANKL and increased immune cell infiltrates leading to peri-implant tissue damage [6, 8, 29, 37]. Specifically, Ti particles inhibited cells comprised in the periodontal ligament (PDL) and alveolar osteogenic cell differentiation by TNF-α, IL-1β, and RANKL [8]. Osteoclast cell precursors including monocytes have been shown to independently differentiate on titanium and aluminum plates [1, 15].

Further disrupting bone resorption and remodeling balances, titanium ions were noted to negatively regulate osteoblast activity by increasing IL-6 and IL-8 expression suggesting a net in vivo result of bone loss by independent and dependent processes such as locally directed immunomodulation [5, 8]. Trace elements within titanium implants have been shown to affect biological systems including DNA synthesis, lipid structure, and proteins such as that in mineralization via alkaline phosphatase mRNA expressions [6, 8, 13]. In bone marrow stem cells (BMSC), Ti particles reportedly promoted increased production of ROS [8]. In the case of DNA synthesis and subsequent damage, mucosal tissue cells were observed to initiate repair under the circumstances [11]. These findings support the in vivo studies of peri-implant bone loss and circumstantial implant failure. Less specifically to dental, orthopedic implant studies have shown titanium ions to be indicative of altered expression of IL-1β, TNF-α, RANKL, and osteoprotegerin (OPG), a protein responsible for inhibition of RANKL activity providing evidence of bone remodeling dysequilibrium [8, 15, 28].

### Septic immune osteolysis

The oral cavity is known to be a habitable environment for many bacterial species which may act harmoniously or detrimental to the host. One of the most commonly found bacteria is Streptococcus mutans. S *mutans*, a facultative anaerobic gram-positive microbe has been correlated with dental caries as well as increased corrosion of implant surfaces by producing an acidogenic environment and LPS which negatively impacts the bioinert titanium surface by inducing electrochemical events along with triggering immune-cellular responses leading to oxidative attack and ion introduction into the peri-implant tissues [14, 15, 38]. Although titanium is not known to be commonly bacteriostatic, studies have shown limited biodiversity of peri-implant flora proportionately to titanium dissolution concentrations, favoring bacteria such as those belonging to the genus Veillonella [14, 39]. In cases of peri-implantitis-related failure, SEM tissue analysis noted a significant reduction of titanium surface concentrations [9]. For the presence of bacteria, debridement of implant surfaces may be warranted despite the. methods utilized to promote surface shedding [28]. In net outcomes, septic-related implant failure may ensue. Debridement can temporarily mitigate bacterial corrosion while conversely introducing titanium into the surrounding tissue suggesting the opening for an alternative material that is less bacterially adhesive and more resistant to corrosion.

## Systemic responses

Mounting evidence has shown titanium nanoparticles in vitro play a role in inducing cellular changes supporting in vivo studies [40]. All metallic products inevitably release particles into the body by means such as galvanization, microbes, biomechanical forces, and tissue/cellular responses [8]. Systems such as cardiovascular, pulmonary, integumentary, digestive, renal, and nervous systems have been shown to by various modes contain titanium dioxide particulates which may produce pathologies in situ [40].

### Cardiovascular

In animal studies, rodents experienced heart damage when exposed to titanium dioxide nanoparticles [40, 41]. Within an in vitro study conducted by *Savi *et al., acute dosing of titanium nanoparticles elicited changes to cardiac excitability. When titanium nanoparticles were administered to mice at an acute dose, cardiac conduction velocity, and tissue excitability increased [42]. *Chen *et al*.* found consistent dosing of titanium nanoparticles led to decreased heart rate and systolic blood pressure while diastolic blood pressure increased [41]. After 90 days of dosing, myocardial injuries were reported, hypothesizing inflammatory responses as being the mediator of this pathology. The results of these studies suggest that titanium induces inflammatory pathways which lead to tissue damage at sites of infiltration.

### Pulmonary/respiratory

Titanium implant placement may cause the release of titanium oxide particles to accumulate in tissues such as lymphatic nodes and pulmonary tissues [3, 4, 6]. Studies and literature regarding TiO₂ nanoparticle responses in pulmonary tissue are connected to increased neutrophil counts as well as cytotoxicity and induced oxidative stress, leading to observed pneumonia [40, 43].

### Integumentary and joints

Case reports have indicated titanium implants to induce dermatological changes [44]. Among affected patients, titanium implants were observed to induce eczema, pruritus, erythema, and vesiculopapular rashes [44, 45]. Due to both commercially pure and alloy titanium implants containing trace elements, there is still conflicting evidence that these dermatological lesions are related directly to titanium particles [45]. Nonetheless, evidence still implies these implants induce allergic skin reactions in select patients.

### Digestive/alimentary system

As previously stated, titanium dioxide is used for many purposes such as material coatings and food additives [46]. In vitro studies with caco-2 cells were used to study the interactions TiO2 nanoparticles would have on the intestinal lumen [46, 47]. The results of these positive nanoparticles on caco-2 monolayers indicated their ability to enter the cell by modes of epithelial growth factor receptor endocytosis and pinocytosis, leading to the expression of NF-κB [46, 47]. Oral administration of titanium dioxide particles in mice resulted in changes in serum ALT/AST and LDH values suggesting reaction formations in hepatocytes. *Wang *et al. found, “hydropic degeneration around the central vein and the spotty necrosis of hepatocytes” to be present upon histopathology examination [48]. The results from these studies suggest another mode along with inhalation which may allow nanoparticles to reach the circulatory system and spread, supporting splenic, hepatic, and renal agglomeration of titanium nanoparticles [40].

### Renal

As mentioned, the introduction of titanium nanoparticles into the bloodstream can lead to accumulations within different organs. Since the kidneys are responsible for regulating serum solutes, their vascular permeability puts them at risk for nephritic inflammation and nanoparticle sequestration [49]. *Gui *et al. found titanium nanoparticles to induce kidney damage by elevation of NF-κB as well as interleukins such as IL-1β, IL-2, IL-6, and IL-10 [49].

### Nervous system

Studies have been published regarding titanium’s ability to pass the blood–brain barrier (BBB) [50, 51]. Like other organs, the effects of titanium nanoparticles on local parenchymal cells induced cellular inflammation, oxidative stress, and ultimately neuronal damage [40, 50, 51]. Elements found within titanium implants such as aluminum may impact neurologic systems and are associated with Alzheimer's, Parkinsons', and amyotrophic lateral sclerosis (ALS) chromium and cobalt are reported to be both genotoxic as well as cytotoxic.

## Zirconia as a viable alternative?

### Zirconia composition

Within the past decade, Zirconia implants have emerged as an alternative to more traditional titanium-based materials. This section aims to evaluate the profile of zirconia implants as a feasible alternative to previous implant materials. Zirconia ceramics are viable dental materials often used for crowns due to their high compressive strength. Typically zirconia is stabilized during firing with the use of yttria (Y2O3) or other stabilizing oxides to increase the physical properties, gaining durability and crack propagation resistance [52–54]. Currently, the standard choice for zirconia implants is composed of a tetragonal poly-crystal, containing 3 mol% yttrium oxide [54]. This material has elicited favorable characteristics for implants due to high flexural strength, low thermal conductivity, resistance to wear, corrosion and fracturing due to zirconias ability to transition into a monoclinic phase so as to prevent propagation of fractures [54].

### Implant healing and osseointegration

Similar to titanium, zirconia displays high reactivity with saliva, resulting in an immediate formation of a dioxide layer preventing corrosion and reducing rates of reaction formation with the implant surface [5]. Tissue healing markers such as NOS1, NOS3, and VEGF1 were significantly lower in soft tissue around zirconia implants compared to the titanium group [35]. Conversely, microvessel density (MVD) reported by *Degidi *et al*.* was reported to be greater in titanium tissues while *Kajiwara *et al*.* suggested improved soft tissue blood flow levels in zirconia abutment groups when compared to titanium abutments [35, 54].

When zirconia was modified with roughened surfaces in animal models, increased wettability, cellular adhesion as well as resulting bone-to-implant contact (BIC) and removal torque values were greater when compared to smoothened zirconia and titanium implant control groups [53, 56–58]. In regards to bone cell proliferation, human lineage osteosarcoma cells were plated on differently modified zirconia surfaces [59]. Herath et al. concluded that roughened zirconia surface modifications induced greater initial surface attachment rates as compared to polished surfaces [59]. In a patient case study, a non-savable zirconia implant due to malpositioning was retrieved and BIC analysis was conducted [9]. The histologic findings showed successful osseointegration further mounting evidence of zirconia as a viable alternative.

#### Immunologic influences

A significant contributor to titanium implant failure was from bacterial influences leading the way to alternative materials to combat the negative impacts of existing materials. *de Oliveira *et al. noted zirconia demonstrating similar pathogenic bacterial adhesion characteristics to alloy titanium surfaces despite zirconia having a lower surface free energy [60]. Although not significant, Aggregatibacter actinomycetemcomitans (A. actinomycetemcomitans) was found to be reduced on zirconia surfaces suggesting a potentially lower bacterial adhesion propensity compared to titanium [60, 61].

Furthermore, recent clinical control trials comparing zirconia to titanium implant survival suggested greater favorability towards zirconia due to lower bacterial loads in collected implant surface samples [61]. Local inflammatory infiltrate was additionally suggested to be significantly lower in zirconia groups than titanium ones [35, 54, 55]. Although lower in zirconia groups, the presence of proinflammatory mediators such as IL-1β and TNF-α were still significantly higher when compared to healthy tissue concentrations [53]. These findings suggest that implant failure from osteolysis due to IL-1β and TNF-α release may be lessened in cases with zirconia versus titanium, potentially providing a stronger prognosis for implant survivability compared to titanium. The oxide surface characteristics of zirconia are not only limited to improved intrinsic cell tolerance but also to demonstrating similar implant surface adhesion of pathogenic bacterial compared to the current gold-standard material of titanium [54, 61, 62].

## Conclusions

The oral cavity is an intense environment for certain materials as heavy mechanical forces, extrinsic molecules, and relatively unstable pH levels induce corrosion. Metal allergies remain difficult to identify and manage. They may pose a significant risk for implant failure and lead to tissue damage and destruction both locally and systemically. Titanium nanoparticles both interact and pass within cellular membranes, promote inflammasome complexes, and release cytokines as well as chemokines. Macroscopically, the effects of titanium nanoparticles are demonstrated in cases where titanium implant failures occur concomitantly with hypersensitivity reactions. Additionally, organ systems are shown to be impacted by the presence of titanium nanoparticles; inducing myocardial damage and pneumonia in live models for example. With commercially pure Titanium, studies showed increased corrosion resistance when acid etched, preventing galvanic coupling and potentially reducing the degree of hypersensitivity in suspected individuals. Patients with titanium allergies often demonstrate co-reactivity with other metals suggesting that a clinician should consider the individual risks and conditions when selecting the appropriate biomaterials for implant placement in the future. It is therefore recommended that if there is any suspicion of a history of metal sensitivity, a lymphocyte transformation test (LTT) may be beneficial so as to choose the proper implant material. As the aim of this article was to briefly investigate the differences in material biocompatibility, further investigation could be done to address the depth of immune impacts both zirconia and titanium may induce. Although many implant complications are attributed to titanium, is important to note that there exists a quantitative data discrepancy of zirconia compared to titanium based implants and that further research must be done to address this matter. There are no statistical indications that specific systemic diseases have significantly increased due to the use of titanium implants, and the risks specific to titanium are not as great as for other metals. Looking forward, it is essential to use a material that builds off of the great qualities that titanium is reputed to have.

## Data Availability

Obtained and examined information utilized in the research process is accessible and provided within the supplementary documents supplemented within this article. Further specifics can be shared upon direct request to the corresponding author.

## Abbreviations

Ti: Titanium
TiO2/TiO: Titanium dioxide
CP: Commercially pure
ROS: Reactive oxygen species
LPS: Lipopolysaccharides
H2S: Hydrogen sulfide
CH3SH: Methanethiol
(CH3)2S: And dimethyl sulfide
APCs: Antigen-presenting cells
TLR: Toll-like receptors
STAT3: Signal transducer and activator of transcription protein 3
NLRP3: NOD-like protein 3
DAMP: Damage-associated molecular pattern
CCR: Chemokine receptors
VEGF: Vascular endothelial growth factor
NOS 1 & 3: Nitric oxide synthase
BCFO: Bone collagen fiber orientations
PDL: Periodontal ligament
BMSCs: Bone marrow stem cells
ALS: Amyotrophic lateral sclerosis
Y2O3: Yttria
MVD: Microvessel density
BIC: Bone to implant contact
LTT: Lymphocyte transformation test
